# Migraine among Patients Presenting With Headaches to the Department of Internal Medicine in a Tertiary Care Centre

**DOI:** 10.31729/jnma.8271

**Published:** 2023-09-30

**Authors:** Alok Dhungel, Asim Pandey, Samriddhi Parajuli, Prajwal Khanal, Nawanit Maskey, Ashaya Luitel

**Affiliations:** 1 Department of Internal Medicine, Kathmandu Medical College and Teaching Hospital, Sinamangal, Kathmandu, Nepal; 2 Kathmandu Medical College and Teaching Hospital, Sinamangal, Kathmandu, Nepal

**Keywords:** *aura*, *headache*, *migraine*, *prevalence*

## Abstract

**Introduction:**

Migraine is one of the common neurovascular disorders characterized by persistent headaches ranging from moderate to severe pain. It has a high prevalence affecting more than one billion population each year across the world and has high morbidity, especially among young adults and females. The exact cause and mechanism of the migraine is unknown. The aim of this study was to find out the prevalence of migraine among patients presenting with headaches to the Department of Internal Medicine in a tertiary care centre.

**Methods:**

A descriptive cross-sectional study was conducted in the Department of Internal Medicine in a tertiary care centre from 1 August 2022 to 31 October 2022. Ethical approval was taken from the Institutional Review Committee. All the patients presenting to the Department of Internal Medicine with headaches as primary complaints were included. Patients with incomplete information and altered sensorium were excluded. Convenience sampling method was used. The point estimate was calculated at a 95% Confidence Interval.

**Results:**

Among 69 patients, the prevalence of migraine was 13 (18.84%) (9.61-28.07, 95% Confidence Interval). Of those diagnosed with migraine headaches, 4 (30.77%) were male patients, and 9 (69.23%) were female patients. The mean age of diagnosis of migraine headache was 30±11.5 years.

**Conclusions:**

The prevalence of migraine among patients presenting with headaches to the Department of Internal Medicine was found to be higher than in the other studies done in similar settings.

## INTRODUCTION

Migraine is a genetically influenced complex primary headache disorder which characterizes episodes of moderate to severe headache, often unilateral and associated with systemic symptoms like nausea, vomiting, phonophobia and/or photophobia. This disorder is often undiagnosed and common cause of disability and loss of work.^1^

Due to its high prevalence with increased morbidity among patients and association with various systemic illnesses, it is considered to be of high public health importance.^2^ The early identification of migraine headaches can help to seek early medical treatment and initiate specific titrated individualized therapy so that it will no longer be a debilitating disease affecting the general population.^3^

The aim of this study was to find out the prevalence of migraine among patients presenting with headaches to the Department of Internal Medicine in a tertiary care centre.

## METHODS

This descriptive cross-sectional study was conducted from 1 August 2022 to 31 October 2022 at Kathmandu Medical College and Teaching Hospital, Sinamangal, Kathmandu, Nepal. Ethical approval was obtained from the Institutional Review Committee (Reference number: 20062022/03). Patients aged 18 to 65 years of age presenting to the Outpatient Department (OPD) or admitted to the Department of internal Medicine were included in the study. Similarly, patients who were not in clear sensorium were not able to participate in the study were excluded in the study. Convenience sampling method was used. The sample size was calculated using the following formula:


$$\begin{array}{ll} \text{n} & ={\text{Z}}^{2}\times \frac{\text{p}\times \text{q}}{{\text{e}}^{\text{2}}}\\ & =1.96^{2}\times \frac{0.12\times 0.88}{0.08^{2}}\\ & =64 \end{array}$$

Where,

n = minimum required sample size
Z = 1.96 at 95% Confidence interval (Ci)
p = prevalence of migraine taken from a previous study, 12%^2^
q = 1-p
e = margin of error, 8%

The minimum sample size calculated was 64. However, the final sample size taken was 69.

The selection and information bias was minimized as possible by collecting data using the pre-designed performa. Details of each participant were filled in by investigators in the OPD or ward with a patient presenting with headache as the primary symptom. Details of their headache characteristics, duration of headache, presence of aura, specific triggers, features and treatment were recorded. The prevalence of migraine was recorded by using a selfreported questionnaire and the severity of migraine headache was calculated using the Migraine Disability Assessment (MIDAS) questionnaire. individual cases of migraine were diagnosed according to international Classification of Headache Disorders (iCHD-3) criteria.^3^

Data was entered and analysed using IBM SPSS Statistics version 24.0. The point estimate was calculated at a 95% Ci.

## RESULTS

Among 69 patients, the prevalence of migraine was 13 (18.84%) (9.61-28.07, 95% Ci). Of those diagnosed with migraine headaches, 4 (30.77%) were male patients, and 9 (69.23%) were female patients. A total of 10 (76.92%) cases of migraine had an aura as an initial symptom. The mean age of diagnosis of migraine headache was 30±11.5 years.

The most common symptom was unilateral headache in about 7 (53.85%) patients and pulsating type of headache in 10 (76.92%) patients. Headache with nausea was seen in 12 (92.31%) and photophobia in 10 (76.92%) patients (Table 1).

**Table 1:** Demographic and clinical features (n= 13).

Characteristics	n (%)
**Gender**	
Male	4 (30.77)
Female	9 (69.23)
**Age group (years)**	
<25	2 (15.38)
25-50	9 (69.23)
>50	2 (15.38)
**Triggering stimulus**	
Stress	10 (76.92)
Hunger	8 (61.54)
Weather	7 (53.85)
Sleep disturbances	6 (46.15)
Lights	6 (46.15)
Perfume	4 (30.77)
Neck pain	4 (30.77)
Alcohol	3 (23.08)
Smoking	3 (23.08)
Hormonal changes	1 (7.67)
**Headache characteristics**	
Pulsating	10 (76.92)
Unilateral	7 (53.85)
Bilateral	5 (38.46)
Heaviness	2 (15.38)
Burning	1 (7.67)
**Associated features**	
Nausea	12 (92.31)
Photophobia	10 (76.92)
Lightheadedness	6 (46.15)
Vomiting	6 (46.15)
Vertigo	7 (53.85)
**Duration of occurrence of symptoms (years)**	
<1	2 (15.38)
1-5	9 (69.23)
>5	2 (15.38)

**Figure 1:**
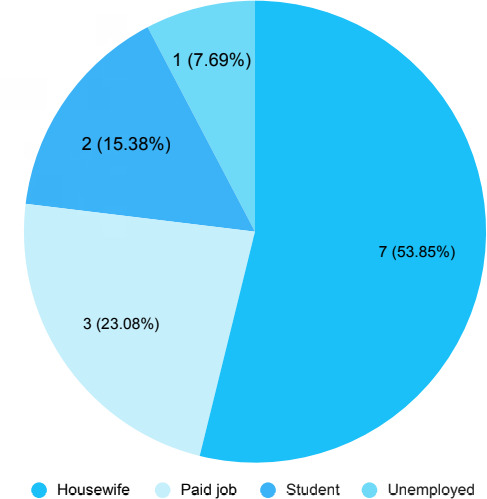
Occupation of the patients (n= 13).

Among them, 5 (38.46%) patients had positive family history. A total of 7 (53.85%) patients worked as a housewife (Figure 1).

A total of 3 (23.07%) patients had moderate disability and 10 (76.92%) had severe disability.

## DISCUSSION

Among 69 patients, the prevalence of migraine was found to be 13 (18.84%). This finding was found to be higher than a study conducted in the past where prevalence was found to be 12%.^2^ Migraine with and without aura, specific triggers, associated symptoms with headache and use of abortive and prophylactic medication were studied using the questionnaire. In our study, the prevalence of migraine was found to be 13 (18.84%). Moreover, according to a study in the United States, the incidence was similar.^4^ However, some studies from India,^5^ China,^6^ and Iran^7^ showed an increased prevalence of migraine among the general population. Due to various reasons such as geography, altitude, cultural differences, and different assessment tools, the incidence of migraine headaches varied across the world.^8^

In our study, we found out that the prevalence of migraine was found to be higher in females 9 (69.23%). This finding was also observed in other studies as well.^3-7^ Migraine often tends to occur during menses in about 60% of women. The loss of estrogen that occurs just prior to menstrual bleeding leads to loss of serotonergic tone in the blood vessels.^8^ This is thought to be one of the triggers for migraine as our study showed 1 (7.67%) of cases of migraine is triggered by hormonal fluctuations.

Results of our current study indicate that the frequency of migraine varies between age groups. Adults aged 25 to 50 are mostly affected by migraine headaches whereas adolescent and old populations are the least affected. This is also consistent with the data published in other studies as well. There might be a multitude of reasons for the difference in incidence based on age. One of the reasons could be increased stress among the adult population. Our study shows that stress 10 (76.92%) is the most common trigger for migraine headaches. Hunger significantly triggers migraine headaches in about 8 (61.54%) of people. Likewise lack of sleep and also flashes of light account for triggering stimulus for a significant amount of cases. Positive family history is present in 5 (38.46%) of cases. The most common clinical feature of migraine headaches is pulsating unilateral headaches preceding with or without aura along with nausea. Our study also showed that 10 (76.92%) of patients had pulsating headaches with unilateral localization in about 7 (53.85%). Therefore, early recognition of symptoms and definitive treatment results in early diagnosis and cure that significantly reduces morbidity among the patients.

The major limitation of this study is that it is a cross-sectional study, so the study data may not be generalizable. Although all the subjects received a physical examination, none of the patients received blood analysis and imaging for diagnosis.

## CONCLUSIONS

The prevalence of migraine among patients presenting with headaches to the Department of Internal Medicine was found to be higher than in the other studies done in similar settings.
